# Transversus Abdominis Plane Block versus Wound Infiltration with Conventional Local Anesthetics in Adult Patients Underwent Surgery: A Systematic Review and Meta-analysis of Randomized Controlled Trials

**DOI:** 10.1155/2020/8914953

**Published:** 2020-03-23

**Authors:** Qiang Cai, Mei-ling Gao, Guan-yu Chen, Ling-hui Pan

**Affiliations:** ^1^Department of Anesthesiology, Guangxi Medical University Cancer Hospital, Nanning, Guangxi 530021, China; ^2^Department of Anesthesiology, Taihe Hospital, Hubei University of Medicine, Shiyan, Hubei 442000, China

## Abstract

**Background:**

How to effectively control the postoperative pain of patients is extremely important to clinicians. Transversus abdominis plane (TAP) block is a novel analgesic method reported to greatly decrease postoperative pain. However, in many areas, there still exists a phenomenon of surgeons using wound infiltration (WI) with conventional local anesthetics (not liposome anesthetics) as the main means to decrease postoperative pain because of traditional wisdom or convenience. Here, we compared the analgesic effectiveness of the two different methods to determine which method is more suitable for adult patients. *Materials and methods*. A systematic review and meta-analysis of randomized controlled trials (RCTs) comparing TAP block and WI without liposome anesthetics in adult patients were performed. Frequently used databases were extensively searched. The main outcomes were postoperative pain scores in different situations (at rest or during movement) and the time until the first use of rescue analgesics. The secondary outcomes were postoperative nausea and vomiting (PONV) incidence and patient satisfaction scores.

**Results:**

Fifteen studies with 983 participants met the inclusion criteria and were included in the present study. The heterogeneity in the final analysis regarding the pain score was low to moderate. The major results of the sensitivity analysis were stable. WI had the same analgesic effect as TAP block only at the one-hour postoperative time point (mean difference = −0.32, 95% confidence interval (-0.87, 0.24), *P* = 0.26) and was associated with a shorter time until the first rescue analgesic and poorer patient satisfaction.

**Conclusion:**

TAP block results in a more effective and steady analgesic effect than WI with conventional local anesthetics in adult patients from the early postoperative period and obtains higher patient satisfaction.

## 1. Introduction

Postoperative severe pain in patients comes mainly from the surgical incision, and visceral tissue damage is a common postoperative problem [1]. Transversus abdominis plane (TAP) block has gradually become an alternative postoperative analgesia technology since Rafi formally described it in 2001 [2], and it has resulted in effective pain relief in operations in which incisions are made in the abdomen [3–6]. However, traditional local anesthetic wound infiltration (WI) by injecting local anesthetics into the incision, favored by surgeons for its convenience, is still the major method used for postoperative analgesia in many areas. Recently, along with the tremendous development of ultrasound techniques, many clinicians have begun to use TAP block and compare it with WI to determine which block is better in adults; nevertheless, the conclusions are still unclear. Therefore, the main purpose of this meta-analysis was to compare the postoperative analgesic effects and safety of TAP block with those of WI without a liposome anesthetic (a type of local anesthetic with an effect for up to 36 h [7]) and the continuous infusion technique in adults after surgeries to obtain a clear conclusion.

## 2. Materials and Methods

### 2.1. Registration

This review followed the Preferred Reporting Items for Systematic Reviews and Meta-Analyses (PRISMA) [8] guidelines ([Supplementary-material supplementary-material-1]) and was registered in the International Prospective Register of Systematic Reviews (PROSPER) (registration number: CRD42019132908).

### 2.2. Literature Search Strategy

The PubMed, Embase, and Cochrane Library databases were systematically searched for randomized controlled trials (RCTs) comparing TAP block with WI from database inception to 1 July 2019. A comprehensive search was performed by combining the free text “Tap,” “Transversus Abdominis,” “Transverse Abdominis,” “Tap block,” and “Transverse Abdominis Plane block” and the Medical Subject Headings (MeSH) term “Transversus Abdominis Plane block” with the free text “Local Anesthesia,” “Infiltration Anesthesia,” “Neural Therapy of Huneke,” “Huneke Neural Therapy,” and “Infiltration” and the MeSH term “Anesthesia, Local.” The only limitation was for human research; no limitation in regard to sex, language, or publication year was applied. The search was independently implemented by two authors (Q.C. and M.L.G.).

### 2.3. Inclusion and Exclusion Criteria

The inclusion criteria were as follows: available, full-text, RCTs comparing TAP block with WI in adults undergoing abdominal surgeries (age greater than 16 years) that included pain scores as pain outcomes after surgery and nausea and vomiting as postoperative complications. The exclusion criteria were as follows: examined a combination of TAP block and WI as the analgesic means, used other nerve blocks as supplementary techniques in or after the operation, used liposomal local anesthetics that were controlled-release drugs for analgesia, or used a continuous infusion technique. Some RCTs did not provide complete information on how they were performed.

### 2.4. Study Selection

Two independent authors (Q.C. and M.L.G.) screened the abstracts and titles of the preliminarily incorporated studies for eligibility according to the inclusion and exclusion criteria. Any disagreements were resolved by discussion between all authors.

### 2.5. Data Collection

A table was created for the data extracted from eligible studies by two independent authors (Q.C. and M.L.G.), and any differences in opinions regarding the data were resolved by discussion with all other authors before the final analysis.

The data items included the characteristics of the trials and patients, details of the interventions and comparators, and the outcomes of the trials. The outcomes we collected included the following: (1) postoperative pain scores at rest and during movement that were rated by the visual analog scale (VAS) or numeric rating scale (NRS) represented as 0 to 10 mm; (2) time to administration of the first rescue analgesic; (3) postoperative nausea and vomiting (PONV) incidence; and (4) satisfaction score of the patients. If the data were presented in another manner or were inadequate, we attempted to e-mail the author to obtain the original data. If we failed to obtain the data, we abandoned the data rather than transforming it to reduce the statistical error.

### 2.6. Statistical Analysis

RevMan 5.3 (The Cochrane Collaboration, Copenhagen, Denmark) was used to address the data analysis. For continuous data, the mean difference (MD) and 95% confidence interval (CI) were used to summarize the data. Dichotomous data are expressed as the risk ratio (RR) with the 95% CI. The heterogeneity of the statistical indicators was tested using *I*^2^ statistics. When the *I*^2^ value was no more than 30%, we regarded the included studies as having acceptable heterogeneity, and the fixed effects model calculated by the Mantel-Haenszel method was used. If the heterogeneity was not low (*I*^2^ > 30%), the random effects model calculated by the DerSimonian and Laird method was used. Forest plots were constructed to show the pooled effects. The diamond in the forest plots represents the pooled effect, and if its 95% CI did not cross the no effect line and its *P* value was <0.05, it was considered statistically significant. In cases of highly significant heterogeneity or to explore whether the type of surgery would affect the pooled result, we conducted subgroup analyses.

### 2.7. Quality Assessment (Risk of Methodological Bias Assessment, Publication Bias, Sensitivity Analysis, and Grading Quality of the Evidence)

The risk of methodological bias in the included studies was assessed using the Cochrane risk of bias tool. Every study included was evaluated by seven parameters, and publication bias was assessed by whether the funnel plots were symmetric. The sensitivity analysis was performed by deleting one study at a time to detect whether the result was stable. We then assessed the quality of the evidence for every outcome with the Grading of Recommendations, Assessment, Development and Evaluations (GRADE) approach [9]. All of the quality assessments were performed by two independent authors (G.Y.C. and C.C.L.), and any disagreements were resolved by discussion with a third author.

## 3. Results

### 3.1. Flowchart of the Literature Search and Study Characteristics

**Figure 1:**
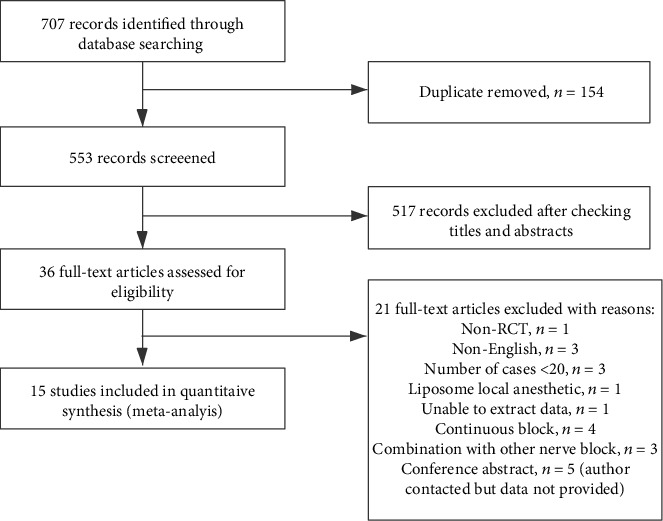
Flowchart of the literature search for the included studies.

A flowchart of the literature search is shown in Figure 1. The preliminary search yielded 707 studies, from which we retained 63 studies for further assessment. Finally, 15 studies including 983 participants were included in our research. The characteristics of the 15 studies are listed in Table 1. The age of the adult participants ranged from 16 to 85 years. All of the studies were selective operations, including general surgeries [10–16], gynecological and obstetric surgeries [17–22], urinary surgeries [23], and nephrology surgeries [24].

### 3.2. Risk of Methodological Bias and the Quality of the Evidence

**Figure 2:**
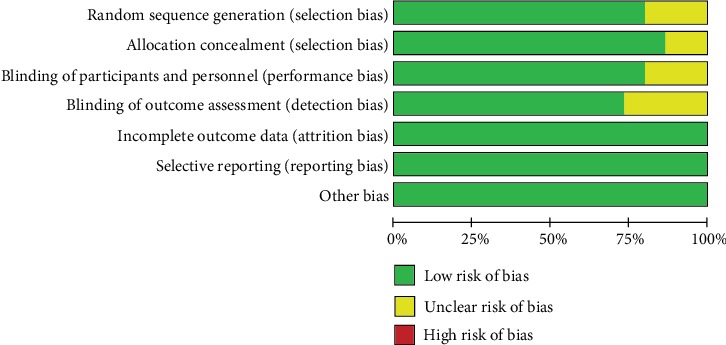
Risk of bias graph for the included studies.

**Figure 3:**
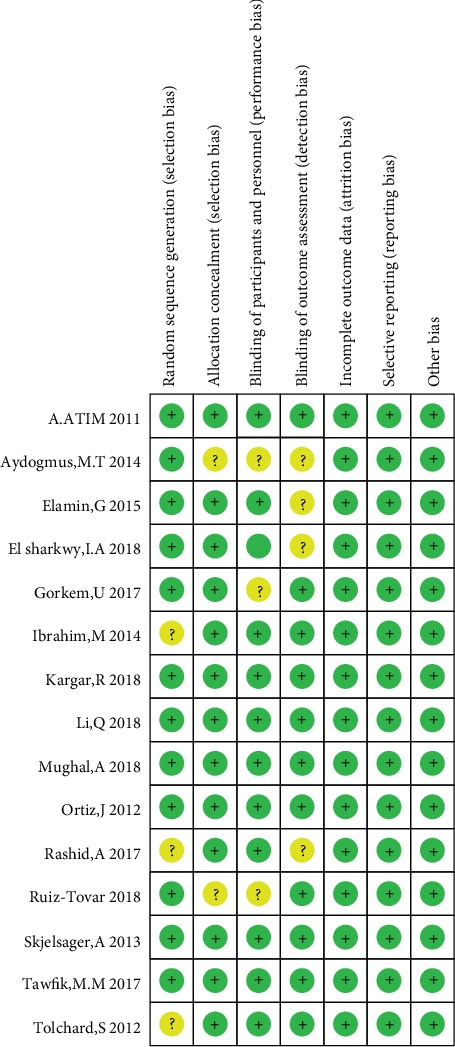
Risk of bias summary for the included studies. Green indicates low risk of bias; yellow indicates unclear risk of bias.

The details of the methodological risk of bias assessment are presented in graphic and summary forms (Figures 2 and 3). In summary, 7 RCTs [10, 15, 17, 20, 22–24] had a low risk of bias, and 8 RCTs [11–14, 16, 18, 19, 21] had an unclear risk of bias. The main reasons for the 8 RCTs having an unclear risk of bias were due to a failure to mention the following factors: randomization sequence generation, allocation concealment, blinding of participants and personnel, and blinding of outcome assessment.

The GRADE evidence profiles for the outcomes were assessed (Tables 2–4). The evidence quality was moderate for pain scores at rest at 1 h, 4 h, 6 h, and 12 h, for pain scores during movement at 4 h, 6 h, and 24 h, and for the time to the first rescue analgesic. The evidence quality was high for pain scores at rest at 2 h and 24 h, for pain scores during movement at 1 h and 2 h, and for PONV incidence and patient satisfaction.

### 3.3. Postoperative Pain Scores at Rest at 1, 2, 4, 6, 12, and 24 H

**Figure 4:**
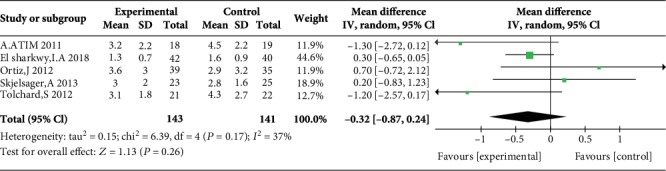
Postoperative pain scores at rest at 1 h.

**Figure 5:**
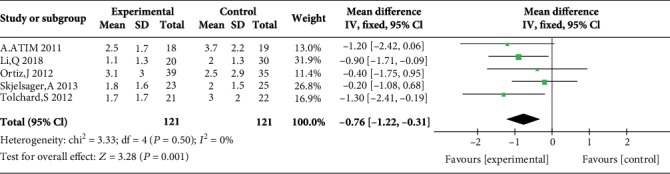
Postoperative pain scores at rest at 2 h.

**Figure 6:**
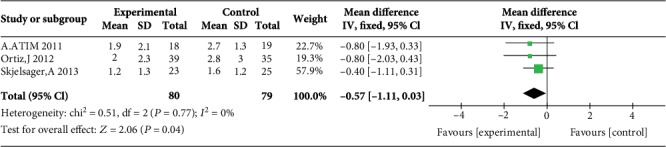
Postoperative pain scores at rest at 4 h.

**Figure 7:**
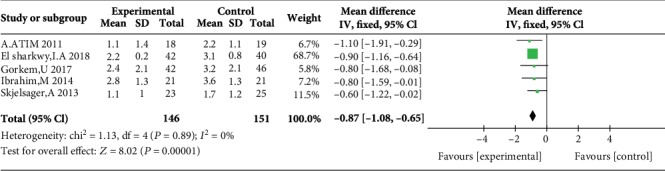
Postoperative pain scores at rest at 6 h.

**Figure 8:**
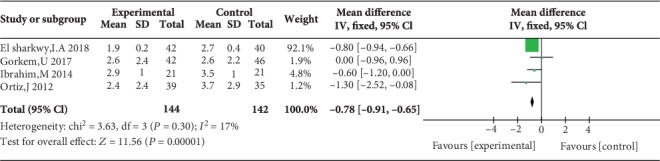
Postoperative pain scores at rest at 12 h.

**Figure 9:**
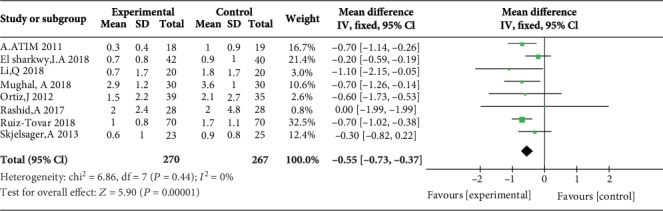
Postoperative pain scores at rest at 24 h.

Five studies reported postoperative pain scores at rest at 1 h [10, 11, 17, 21, 23], five studies reported postoperative pain scores at rest at 2 h [10, 11, 17, 23, 24], three studies reported postoperative pain scores at rest at 4 h [10, 17, 23], five studies reported postoperative pain scores at rest at 6 h [12, 17, 19, 21, 23], four studies reported postoperative pain scores at rest at 12 h [10, 12, 19, 21], and eight studies reported postoperative pain scores at rest at 24 h [10, 14–17, 21, 23, 24]. Compared with WI, TAP was associated with lower pain scores at rest at 2 h (MD = −0.76, 95% CI (-1.22, 0.31), *P* = 0.001), 4 h (MD = −0.57, 95% CI (-1.11, 0.03), *P* = 0.04), 6 h (MD = −0.87, 95% CI (-1.08, 0.65), *P* < 0.00001), 12 h (MD = −0.78, 95% CI (-0.91, 0.65), *P* < 0.00001), and 24 h (MD = −0.55, 95% CI (-0.73, 0.37), *P* < 0.00001) but not at 1 h (MD = −0.32, 95% CI (-0.87, 0.24), *P* = 0.26), and there were low to moderate levels of heterogeneity in six analyses (for 1 h: *I*^2^ = 37%; for 2 h: *I*^2^ = 0%; for 4 h: *I*^2^ = 0%; for 6 h: *I*^2^ = 0%; for 12 h: *I*^2^ = 17%; and for 24 h: *I*^2^ = 0%) (Figures 4–9). Furthermore, to explore whether the different types of surgery had an impact on the pooled results, we carried out subgroup analyses. In the subgroup analyses of nonlaparoscopic surgery, compared with WI, TAP block was associated with lower pain scores at rest at 2 h (MD = −0.69, 95% CI (-1.23, -0.16), *I*^2^ = 4%), 6 h (MD = −0.79, 95% CI (-1.22, -0.36), *I*^2^ = 0%), and 24 h (MD = −0.58, 95% CI (-0.90, -0.26), *I*^2^ = 15%) but not at 1 h (MD = −0.32, 95% CI (-1.15, -0.52), *I*^2^ = 64%), and in the subgroup analyses of laparoscopic surgery, compared with WI, TAP block was also associated with lower pain scores at rest at 2 h (MD = −0.94, 95% CI (-1.79, -0.08), *I*^2^ = 2%), 6 h (MD = −0.89, 95% CI (-1.13, -0.65), *I*^2^ = 0%), and 24 h (MD = −0.53, 95% CI (-0.75, -0.31), *I*^2^ = 10%) but not at 1 h (MD = −0.30, 95% CI (-0.63, 0.03), *I*^2^ = 44%) ([Supplementary-material supplementary-material-1] to [Supplementary-material supplementary-material-1]). Moreover, in the subgroup analyses of the surgical site in the upper abdomen, compared with WI, TAP block was associated with lower pain scores at rest at 2 h (MD = −0.94, 95% CI (-1.79, -0.08), *I*^2^ = 2%), 12 h (MD = −0.74, 95% CI (-1.28, -0.20), *I*^2^ = 1%), and 24 h (MD = −0.69, 95% CI (-1.00, -0.39), *I*^2^ = 0%) but not at 1 h (MD = −0.26, 95% CI (-2.12, -1.60), *I*^2^ = 72%), and in the subgroup analyses of surgical site in the lower abdomen, compared with WI, TAP block was also associated with lower pain scores at rest at 2 h (MD = −0.69, 95% CI (-1.23, -0.16), *I*^2^ = 4%), 12 h (MD = −0.78, 95% CI (-0.92, -0.65), *I*^2^ = 61%), and 24 h (MD = −0.47, 95% CI (-0.69, -0.24), *I*^2^ = 9%) but not at 1 h (MD = −0.32, 95% CI (-0.86, 0.22), *I*^2^ = 29%) ([Supplementary-material supplementary-material-1] to [Supplementary-material supplementary-material-1]).

### 3.4. Postoperative Pain Scores during Movement at 1, 2, 4, 6, 12, and 24 H

**Figure 10:**
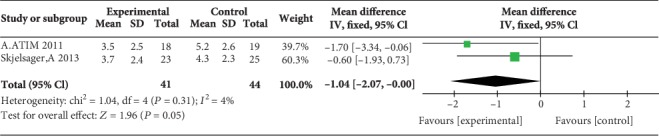
Postoperative pain scores during movement at 1 h.

**Figure 11:**
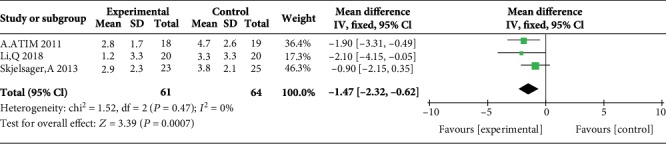
Postoperative pain scores during movement at 2 h.

**Figure 12:**
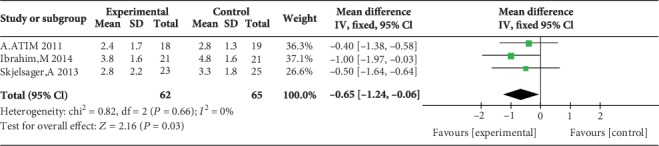
Postoperative pain scores during movement at 4 h.

**Figure 13:**
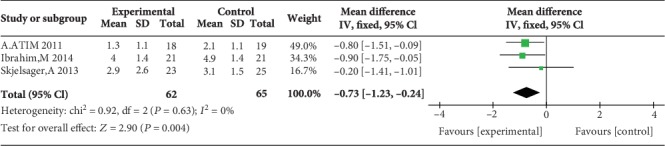
Postoperative pain scores during movement at 6 h.

**Figure 14:**
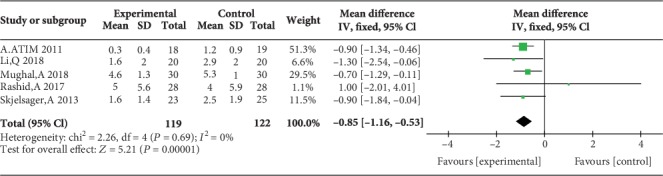
Postoperative pain scores during movement at 24 h.

Two studies reported postoperative pain scores during movement at 1 h [17, 23], three studies reported postoperative pain scores during movement at 2 h [17, 23, 24], three studies reported postoperative pain scores during movement at 4 h [12, 17, 23], three studies reported postoperative pain scores during movement at 6 h [12, 17, 23], no study reported postoperative pain scores during movement at 12 h, and five studies reported postoperative pain scores during movement at 24 h [14, 15, 17, 23, 24]. Compared with WI, TAP block was associated with lower pain scores during movement at 2 h (MD = −1.47, 95% CI (-2.32, 0.62), *P* = 0.0007), 4 h (MD = −0.65, 95% CI (-1.24, 0.06), *P* = 0.03), 6 h (MD = −0.73, 95% CI (-1.23, 0.24), *P* = 0.004), and 24 h (MD = −0.85, 95% CI (-1.16, 0.53), *P* < 0.00001) but not at 1 h (MD = −1.04, 95% CI (-2.07, 0.00), *P* = 0.05), and there were low levels of heterogeneity in five analyses (for 1 h: *I*^2^ = 4%; for 2 h: *I*^2^ = 0%; for 4 h: *I*^2^ = 0%; for 6 h: *I*^2^ = 0%; and for 24 h: *I*^2^ = 0%) (Figures 10–14). Because the number of studies reporting pain scores during movement at 1, 2, 4, 6, and 12 h was no more than 3, we did not conduct a subgroup analysis on pain scores during movement.

### 3.5. Time until the First Rescue Analgesic

**Figure 15:**
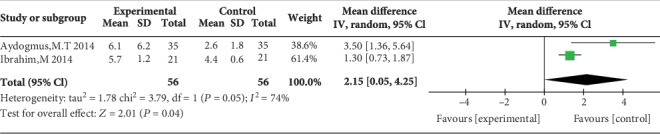
Time to the first rescue analgesic.

Two studies reported the time until the first rescue analgesic, and the overall effect of the pooled studies showed that the time to the first rescue analgesic in the TAP block group was longer than that in the WI group (MD = 2.15, 95% CI (0.05, 4.25), *P* = 0.04). However, the heterogeneity was high (*I*^2^ = 74%) (Figure 15).

### 3.6. PONV Incidence and Patient Satisfaction

**Figure 16:**
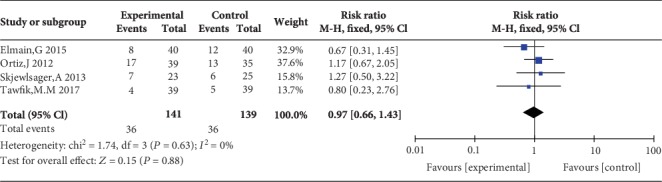
Postoperative nausea and vomiting (PONV) incidence.

**Figure 17:**
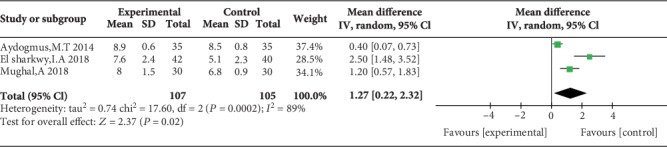
Patient satisfaction.

Four studies reported PONV incidence [10, 13, 20, 23], and the overall effect of the pooled studies showed that PONV incidence was not different between groups (OR = 0.97, 95% CI (0.66, 1.43), *P* = 0.88), and the heterogeneity was low (*I*^2^ = 0%) (Figure 16). Three studies reported that patient satisfaction with TAP was higher than that with WI (MD = 1.27, 95% CI (0.22, 2.32), *P* = 0.02), but the heterogeneity was high (*I*^2^ = 89%) (Figure 17).

### 3.7. Publication Bias and Sensitivity Analysis

**Figure 18:**
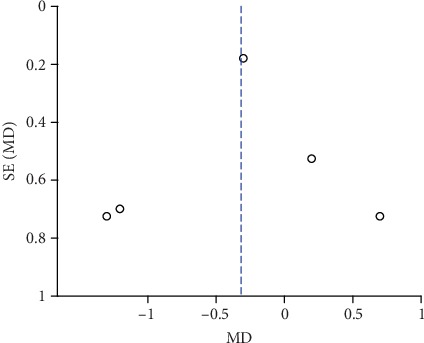
Funnel plot of pain scores at rest at 1 h.

**Figure 19:**
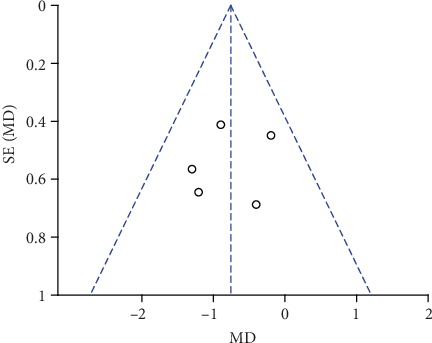
Funnel plot of pain scores at rest at 2 h.

**Figure 20:**
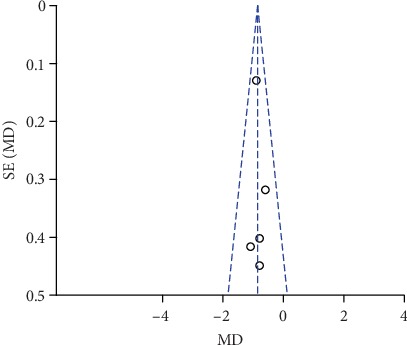
Funnel plot of pain scores at rest at 6 h.

**Figure 21:**
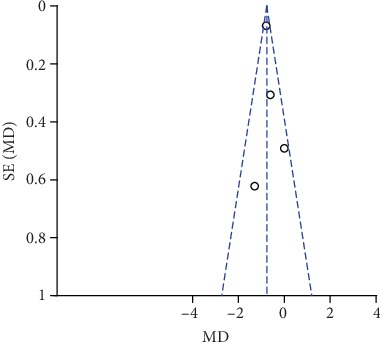
Funnel plot of pain scores at rest at 12 h.

**Figure 22:**
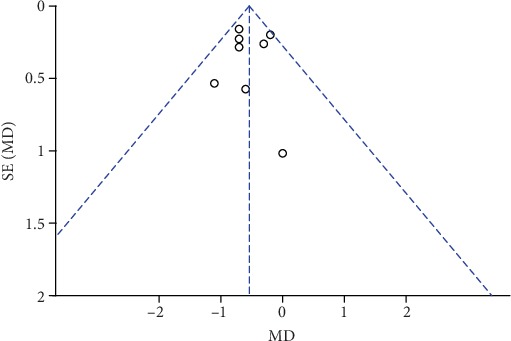
Funnel plot of pain scores at rest at 24 h.

**Figure 23:**
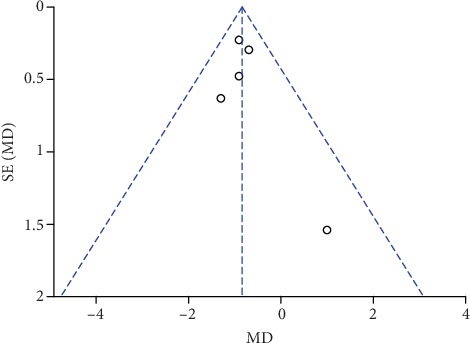
Funnel plot of pain scores during movement at 24 h.

**Figure 24:**
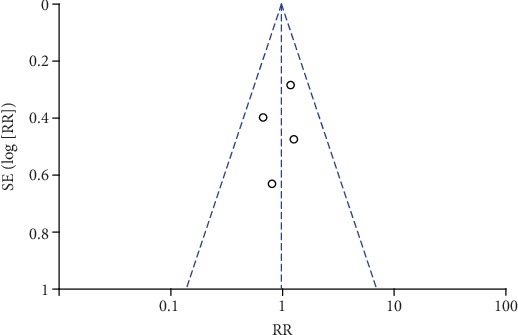
Funnel plot of PONV incidence.

The funnel plots of pain scores at rest at 1 h, 2 h, 6 h, 12 h, and 24 h (Figures 18–22) and during movement at 24 h (Figure 23) and the funnel plots of PONV incidence (Figure 24) were symmetric, indicating no or slight publication bias. Since the number of included studies that reported pain scores at rest at 4 h or during movement at 1 h, 2 h, 4 h, 6 h, and 12 h was less than 3, we did not draw funnel plots. A similar situation also occurred in the funnel plots of the time until the first rescue analgesic and patient satisfaction. We performed a sensitivity analysis of the overall effects of the pooled studies on the pain score. When we deleted one study at a time, the overall effects of the pooled studies on the pain scores at most time points were consistent with those before exclusion, which suggested a stable result; however, the overall effects of the pooled studies on the pain scores at rest at 4 h and during movement at 1 h, 4 h, and 6 h varied to the contrary compared with those before exclusion, possibly because few studies included this particular time point (no more than 3). The results of the sensitivity analysis are shown in Table 5.

## 4. Discussion

This is the first meta-analysis to compare the analgesic effect and safety of TAP block with those of WI using conventional local anesthetics in adult patients. After assessing 15 studies with 983 patients, the final results indicated that WI had the same analgesic effect as TAP block in a short postoperative period (only one hour), with moderate evidence (as evaluated by GRADE), resulted in a shorter time to the initial rescue analgesic, with moderate evidence, and had poorer patient satisfaction and similar PONV incidence, with high evidence.

Specifically, except for the pain scores at 1 h after the operation, significant differences were found at 2, 4, 6, 12, and 24 h, and the heterogeneity at all time points was low to moderate. Moreover, the results of the sensitivity analysis were stable except for several time points when few studies were included. Further subgroup analyses of the effect of the type of operation on pain scores indicated that the laparoscopic and nonlaparoscopic surgery subgroups had no difference in pain scores between TAP block and WI at any time point; moreover, there was no difference between upper abdominal surgery and lower abdominal surgery. All of these results suggest that WI might display much shorter analgesic action than TAP block with conventional local anesthetics after abdominal surgery, as evidenced by similar postoperative analgesic effects only at one hour after surgery. Interestingly, many studies have reported that WI with conventional local anesthetics decreased postoperative pain scores over only a very short time, even compared with saline. In a study that included 260 women undergoing breast surgery, Albi-Feldzer et al. found that the WI group had a lower score than the saline group in the first 90 min after the end of surgery [25]. Abbas et al. found that between the WI and saline groups, there was no difference at 4 h after the operation in patients undergoing laparoscopic total extraperitoneal repair of unilateral inguinal hernias [26]. The neglected reason for the short duration of action of WI may be that WI could result in rapid drug absorption because the local anesthetics were not injected into the space between the muscles that contains abundant nerve branches [27] but rather into the muscular tissue, which is rich in blood capillaries and can accelerate drug absorption. However, in TAP block, local anesthetics are injected into the space between the transversus abdominis and internal oblique muscles [3], where thoracolumbar nerves run from the T6 to L1 spinal roots, which control the sense of the whole anterolateral abdominal wall [27], making the block more efficient.

As evidence suggests, the use of continuous catheter technology [28, 29] could extend the analgesic duration of WI; a meta-analysis including 29 RCTs containing 2059 patients showed that continuous WI with preperitoneal wound catheters was as effective method as epidural analgesia (which is a valid method) in pain control after abdominal surgery [30]. Correspondingly, the pooled result for the time to the first rescue analgesic was shorter in the WI group than that in our study.

Nausea and vomiting are common complications and frequently occur after surgery [31]; this phenomenon is known as PONV. Until now, the mechanisms underlying this outcome have been unclear [32]. The studies included in the present meta-analysis reported no differences between TAP block and WI. In fact, many studies comparing TAP or WI with placebo did not indicate significant changes in PONV incidence. A meta-analysis of 56 studies found no significant differences between TAP block, placebo or no block, and epidural analgesia [33]. Similarly, there were no differences between WI and placebo in PONV incidence for postcesarean section analgesia in a meta-analysis that included 21 studies [34]. In addition, some individuals are concerned that local anesthetics being absorbed into the blood at different rates in TAP block and WI might influence PONV incidence. However, according to data on the risk factors for PONV [31], the local anesthetics used in TAP block or WI are not high-risk drug factors, which include volatile anesthetics, nitrous oxide, and intraoperative opioids.

Finally, we found in the present meta-analysis that TAP block resulted in higher patient satisfaction than WI, and for the few studies included, the heterogeneity for this analysis was high. Therefore, additional RCTs should be conducted in the future to verify this hypothesis.

However, there were many limitations to our meta-analysis. First, it was impossible to obtain all the data for the included studies. Some studies [35, 36] used quartiles to represent the data, which could not be accurately converted to the mean plus standard deviation. We tried to contact the authors to acquire primary data but failed. To reduce methodological heterogeneity, we did not include these studies. Second, although inclusion criteria were applied, there still existed heterogeneity that might have been due to different types of surgeries and anesthetics, the time and types of interventions, and the concentrations and volumes of anesthetics; however, limited by the number of studies included, we could not carry out a more detailed subgroup analysis (e.g., subgroup analysis of the surgical category), and the sensitivity analysis of the few results was not stable; hence, more RCTs are needed for further study. Third, initially, we did not include studies on child participants [37–42] in our meta-analysis; however, this does not signify that this question in children is not important to our clinical research. According to recent research on the use of TAP block in children, the use of TAP block seemed to produce favorable clinical effects. Fourth, because of the limitations of RevMan software, we could not perform a quantitative analysis of publication bias (e.g., Begger's test or Egger's test), but funnel plots of most results did not show publication bias. In future research, we should use more accurate tools for analysis.

## 5. Conclusion

In conclusion, our review of moderate evidence supports the notion that TAP block can result in more effective analgesia than WI using conventional local anesthetics in adult patients from the early postoperative period and acquire higher patient satisfaction regardless of laparoscopic surgery or nonlaparoscopic surgery or upper abdominal surgery or lower abdominal surgery.

## Figures and Tables

**Table 1:** Characteristics of the included studies.

Author (publication year)	Age of patients (years)	Surgery	Anesthesia	Number of patients	Technology of TAP block/WI	Drug	Operative site
Atim, A. [17] (2011)	Adults (30-63)	Hysterectomy	GA	TAP (18), WI (19)	US-guided bilateral injection/incision site infiltration	Bupivacaine	Lower abdomen
Ortiz, J. [10] (2012)	Adults (18-64)	Laparoscopic cholecystectomy	GA	TAP (39), WI (35)	US-guided bilateral injection/trocar insertion site infiltration	Bupivacaine	Upper abdomen
Tolchard, S. [11] (2012)	Adults (>16)	Laparoscopic cholecystectomy	GA	TAP (21), WI (22)	US-guided bilateral injection//trocar insertion site infiltration	Bupivacaine	Upper abdomen
Skjelsager, A. [23] (2013)	Adults (18-80)	Open radical prostatectomy	GA	TAP (23), WI (25)	US-guided bilateral injection/incision site infiltration	Bupivacaine	Lower abdomen
Aydogmus, M. T. [18] (2014)	Pregnant women (23-35)	Cesarean delivery	GA	TAP (35), WI (35)	US-guided bilateral injection/incision site infiltration	Levobupivacaine	Lower abdomen
Ibrahim, M. [12] (2014)	Adults (>18)	Laparoscopic sleeve gastrectomy	GA	TAP (21), WI (21)	US-guided bilateral injection/trocar insertion site infiltration	Bupivacaine	Upper abdomen
Elamin, G. [13] (2015)	Adults (18-85)	Elective laparoscopic cholecystectomy	GA	TAP (40), WI (40)	Laparoscope-guided bilateral injection/trocar insertion site infiltration	Bupivacaine	Upper abdomen
Gorkem, U. [19] (2017)	Pregnant women (18-45)	Cesarean delivery	GA	TAP (42), WI (46)	US-guided bilateral injection/incision site infiltration	Bupivacaine	Lower abdomen
Rashid, A. [14] (2017)	Adults (>18)	Elective laparoscopic colonic surgery	GA	TAP (28), WI (28)	US-guided bilateral injection/trocar insertion site infiltration	Bupivacaine	Lower abdomen
Tawfik, M. M. [20] (2017)	Pregnant women (22-31)	Cesarean delivery	SA	TAP (39), WI (39)	US-guided bilateral injection/incision site infiltration	Bupivacaine	Lower abdomen
El sharkwy, I. A. [21] (2018)	Women (>18)	Gynecologic laparoscopy	GA	TAP (42), WI (40)	US-guided bilateral injection/trocar insertion site infiltration	Bupivacaine	Lower abdomen
Kargar, R. [22] (2018)	Adults (18-50)	Laparoscopic excision of endometriosis	GA	TAP (24), WI (21)	US-guided bilateral injection/trocar insertion site infiltration	Bupivacaine	Lower abdomen
Li, Q. [24] (2018)	Adults (18-75)	Peritoneal dialysis catheter implantation	SA	TAP (20), WI (20)	US-guided unilateral injection/incision site infiltration	Ropivacaine	Lower abdomen
Mughal, A. [15] (2018)	Adults (18-80)	Total extraperitoneal inguinal hernia repair	GA	TAP (30), WI (30)	Laparoscope-guided unilateral injection/incision site infiltration	Bupivacaine	Lower abdomen
Ruiz-Tovar [16] (2018)	Adults (41-48)	Laparoscopic Roux-en-Ygastric bypass	GA	TAP (70), WI (70)	Laparoscope-guided bilateral injection/trocar insertion site infiltration	Bupivacaine	Upper abdomen

GA: general anesthesia; SA: spinal epidural anesthesia; TAP: transversus abdominis plane; WI: wound infiltration; US: ultrasound.

**Table 2:** GRADE evidence profile for pain scores.

Quality assessment							No. of patients		Effect	Quality	Importance
No. of studies	Design	Risk of bias	Inconsistency	Indirectness	Imprecision	Other considerations	TAP	Wl	Absolute (95% CI)		
Pain scores at rest at 1 h (measured with: VAS; better indicated by lower values)											
5	Randomized trials	Serious^1^	No serious inconsistency	No serious indirectness	Serious^2^	Strong association^3^	143	141	MD 0.32 lower (0.87 lower to 0.24 higher)	Moderate	Critical
Pain scores at rest at 2 h (measured with: VAS; better indicated by lower values)											
5	Randomized trials	No serious	No serious inconsistency	No serious indirectness	Serious^4^	Strong association^3^	121	121	MD 0.76 lower (1.22 to 0.31 lower)	High	Critical
Pain scores at rest at 4 h (measured with: VAS; better indicated by lower values)											
3	Randomized trials	No serious	No serious inconsistency	No serious indirectness	Serious^2^	None	80	79	MD 0.57 lower (1.11 to 0.03 lower)	Moderate	Critical
Pain scores at rest at 6 h (measured with: VAS; better indicated by lower values)											
5	Randomized trials	Serious^5^	No serious inconsistency	No serious indirectness	Serious^2^	Strong association^3^	146	151	MD 0.87 lower (1.08 to 0.65 lower)	Moderate	Critical
Pain scores at rest at 12 h (measured with: VAS; better indicated by lower values)											
4	Randomized trials	Serious^1^	No serious inconsistency	No serious indirectness	Serious	Strong association^3^	144	142	MD 0.78 lower (0.91 to 0.65 lower)	Moderate	Critical
Pain scores at rest at 24 h (measured with: VAS; better indicated by lower values)											
8	Randomized trials	Serious^6^	No serious inconsistency	No serious indirectness	Serious^2^	Very strong association^7^	270	267	MD 0.55 lower (0.73 to 0.37 lower)	High	Critical
Pain scores during movement at 1 h (measured with: VAS; better indicated by lower values)											
2	Randomized trials	No serious	No serious inconsistency	No serious indirectness	No serious imprecision	None	41	44	MD 1.04 lower (2.07 lower to 0 higher)	High	Critical
Pain scores during movement at 2 h (measured with: VAS; better indicated by lower values)											
3	Randomized trials	No serious	No serious inconsistency	No serious indirectness	No serious imprecision	None	61	64	MD 1.47 lower (2.32 to 0.62 lower)	High	Critical
Pain scores during movement at 4 h (measured with: VAS; better indicated by lower values)											
3	Randomized trials	No serious	No serious inconsistency	No serious indirectness	Serious^5^	None	62	65	MD 0.65 lower (1.24 to 0.06 lower)	Moderate	Critical
Pain scores during movement at 6 h (measured with: VAS; better indicated by lower values)											
3	Randomized trials	No serious	No serious inconsistency	No serious indirectness	Serious^6^	None	62	65	MD 0.73 lower (1.23 to 0.24 lower)	Moderate	Critical
Pain scores during movement at 24 h (measured with: VAS; better indicated by lower values)											
5	Randomized trials	Serious^6^	No serious inconsistency	No serious indirectness	Serious^2^	Strong association^3^	119	122	MD 0.85 lower (1.16 to 0.53 lower)	Moderate	Critical

^1^One study did not mention the randomization method, and two studies did not mention how the blindness method was implemented.

^2^Some data were collected from charts by a measurement tool or converted by a formula.

^3^More than 200 patients were enrolled.

^4^One study did not mention the randomization method, and one study did not mention how the blindness method was implemented.

^5^Several studies did not mention the randomization and blindness methods.

^6^One study did not mention the randomization and blindness methods.

^7^More than 500 patients were enrolled.

**Table 3:** GRADE evidence profile for the time to the first rescue analgesic.

Quality assessment							No. of patients		Effect	Quality	Importance
No. of studies	Design	Risk of bias	Inconsistency	Indirectness	Imprecision	Other considerations	TAP	Wl	Absolute (95% CI)		
Time to the first rescue analgesic (better indicated by lower values)											
2	Randomized trials	Serious^1^	No serious inconsistency	No serious indirectness	No serious imprecision	None	56	56	MD 2.15 higher (0.05 to 4.25 higher)	Moderate	Important

^1^Some studies did not mention the randomization or blindness method.

**Table 4:** GRADE evidence profile for postoperative nausea and vomiting (PONV) incidence and patient satisfaction.

Quality assessment							No. of patients		Effect		Quality	Importance
No. of studies	Design	Risk of bias	Inconsistency	Indirectness	Imprecision	Other considerations	TAP	Wl	Absolute (95% CI)			
Postoperative nausea and vomiting (PONV) incidence												
4	Randomized trials	No serious	No serious inconsistency	No serious indirectness	No serious imprecision	None	36/141 (25.5%)	36/139 (25.9%)	RR 0.97 (0.66 to 1.43)	8 fewer per 1000 (from 88 fewer to 111 more)	High	Important
								27%		8 fewer per 1000 (from 92 fewer to 116 more)		
Patient satisfaction (better indicated by lower values)												
3	Randomized trials	No serious	No serious inconsistency	No serious indirectness	No serious imprecision	None	107	105	—	MD 1.27 higher (0.22 to 2.32 higher)	High	Critical

**Table 5:** Sensitivity analysis of the pain score.

Pain score statistics with each study removed					
Study	MD	95% CI lower limit	95% CI upper limit	*Z* value	*P* value
TAP VS WI at rest at 1 h					
Atim, A. [17] (2011)	-0.20	-0.74	0.35	0.71	0.48
El sharkwy, I. A. [21] (2018)	-0.36	-1.31	0.58	0.75	0.45
Ortiz, J. [10] (2012)	-0.44	-0.98	0.10	1.59	0.11
Skjelsager, A. [23] (2013)	-0.46	-1.16	0.24	1.29	0.20
Tolchard, S. [11] (2012)	-0.20	-0.76	0.37	0.67	0.50
TAP VS WI at rest at 2 h					
Atim, A. [17] (2011)	-0.70	-1.18	-0.21	2.8	0.005
Li, Q. [24] (2018)	-0.70	-1.25	-0.15	2.48	0.01
Ortiz, J. [10] (2012)	-0.81	-1.29	-0.32	3.28	0.001
Skjelsager, A. [23] (2013)	-0.97	-1.50	-0.44	3.56	0.0004
Tolchard, S. [11] (2012)	-0.65	-1.15	-0.15	2.56	0.01
TAP VS WI at rest at 4 h					
Atim, A. [17] (2011)	-0.50	-1.11	0.11	1.60	0.11
Ortiz, J. [10] (2012)	-0.51	-1.11	0.09	1.67	0.09
Skjelsager, A. [23] (2013)	-0.80	-1.63	0.03	1.88	0.06
TAP VS WI at rest at 6 h					
Atim, A. [17] (2011)	-0.85	-1.07	-0.63	7.6	<0.00001
El sharkwy, I. A. [21] (2018)	-0.79	-1.17	-0.41	4.1	<0.0001
Gorkem, U. [19] (2017)	-0.87	-1.09	-0.65	7.82	<0.00001
Ibrahim, M. [12] (2014)	-0.87	-1.09	-0.65	7.77	<0.00001
Skjelsager, A. [23] (2013)	-0.90	-1.13	-0.68	7.85	<0.00001
TAP VS WI at rest at 12 h					
El sharkwy, I. A. [21] (2018)	-0.56	-1.03	-0.09	2.33	0.02
Gorkem, U. [19] (2017)	-0.80	-0.93	-0.66	11.67	<0.00001
Ibrahim, M. [12] (2014)	-0.79	-0.93	-0.65	11.42	<0.00001
Ortiz, J. [10] (2012)	-0.78	-0.91	-0.64	11.41	<0.00001
TAP VS WI at rest at 24 h					
Atim, A. [17] (2011)	-0.52	-0.72	-0.32	5.08	<0.00001
El sharkwy, I. A. [21] (2018)	-0.64	-0.85	-0.44	6.13	<0.00001
Li, Q. [24] (2018)	-0.53	-0.71	-0.35	5.63	<0.00001
Mughal, A. [15] (2018)	-0.53	-0.72	-0.34	5.39	<0.00001
Ortiz, J. [10] (2012)	-0.55	-0.73	-0.36	5.81	<0.00001
Rashid, A. [14] (2017)	-0.55	-0.73	-0.37	5.92	<0.00001
Ruiz-Tovar [16] (2018)	-0.47	-0.69	-0.25	4.19	<0.0001
Skjelsager, A. [23] (2013)	-0.58	-0.78	-0.39	5.87	<0.00001
TAP VS WI during movement at 1 h					
Atim, A. [17] (2011)	-0.60	-1.93	0.73	0.88	0.38
Skjelsager, A. [23] (2013)	-1.70	-3.34	-0.06	2.03	0.04
TAP VS WI during movement at 2 h					
Atim, A. [17] (2011)	-1.23	-2.29	-0.16	2.25	0.02
Li, Q. [24] (2018)	-1.34	-2.28	-0.41	2.81	0.005
Skjelsager, A. [23] (2013)	-1.96	-3.12	-0.80	3.32	0.0009
TAP VS WI during movement at 4 h					
Atim, A. [17] (2011)	-0.79	-1.53	-0.05	2.10	0.04
Ibrahim, M. [12] (2014)	-0.44	-1.19	0.30	1.17	0.24
Skjelsager, A. [23] (2013)	-0.70	-1.39	-0.02	2.00	0.05
TAP VS WI during movement at 6 h					
Atim, A. [17] (2011)	-0.67	-1.37	0.02	1.89	0.06
Ibrahim, M. [12] (2014)	-0.65	-1.26	-0.04	2.07	0.04
Skjelsager, A. [23] (2013)	-0.84	-1.38	-0.30	3.03	0.002
TAP VS WI during movement at 24 h					
Atim, A. [17] (2011)	-0.79	-1.25	-0.33	3.39	0.0007
Li, Q. [24] (2018)	-0.81	-1.14	-0.48	4.84	<0.00001
Mughal, A. [15] (2018)	-0.91	-1.29	-0.53	4.69	<0.00001
Rashid, A. [14] (2017)	-0.87	-1.19	-0.55	5.30	<0.00001
Skjelsager, A. [23] (2013)	-0.84	-1.18	-0.50	4.86	<0.00001

## Data Availability

The data supporting this systematic review or meta-analysis were obtained from previously reported studies and datasets that have been cited. The processed data can be acquired from the corresponding author upon request.

## Supplementary Materials

Supplementary 1 Table S1: PRISMA checklist (DOC).

Supplementary 2 Fig S1: subgroup analysis of pain scores at rest at 1 h after nonlaparoscopic surgery VS laparoscopic surgery (TIF).

Supplementary 3 Fig S2: subgroup analysis of pain scores at rest at 2 h after nonlaparoscopic surgery VS laparoscopic surgery (TIF).

Supplementary 4 Fig S3: subgroup analysis of pain scores at rest at 6 h after nonlaparoscopic surgery VS laparoscopic surgery (TIF).

Supplementary 5 Fig S4: subgroup analysis of pain scores at rest at 24 h after nonlaparoscopic surgery VS laparoscopic surgery (TIF).

Supplementary 6 Fig S5: subgroup analysis of pain scores at rest at 1 h after upper abdominal surgery VS lower abdominal surgery (TIF).

Supplementary 7 Fig S6: subgroup analysis of pain scores at rest at 2 h after upper abdominal surgery VS lower abdominal surgery (TIF).

Supplementary 8 Fig S7: subgroup analysis of pain scores at rest at 12 h after upper abdominal surgery VS lower abdominal surgery (TIF).

Supplementary 9 Fig S8: subgroup analysis of pain scores at rest at 24 h after upper abdominal surgery VS lower abdominal surgery (TIF).
