# Supplementary figures and images for: Transversus Abdominis Plane Block versus Wound Infiltration with Conventional Local Anesthetics in Adult Patients Underwent Surgery: A Systematic Review and Meta-analysis of Randomized Controlled Trials
Source: Biomed Res Int. 2020 Mar 23;2020:8914953. doi: 10.1155/2020/8914953 (PMC7125448; doi:10.1155/2020/8914953)

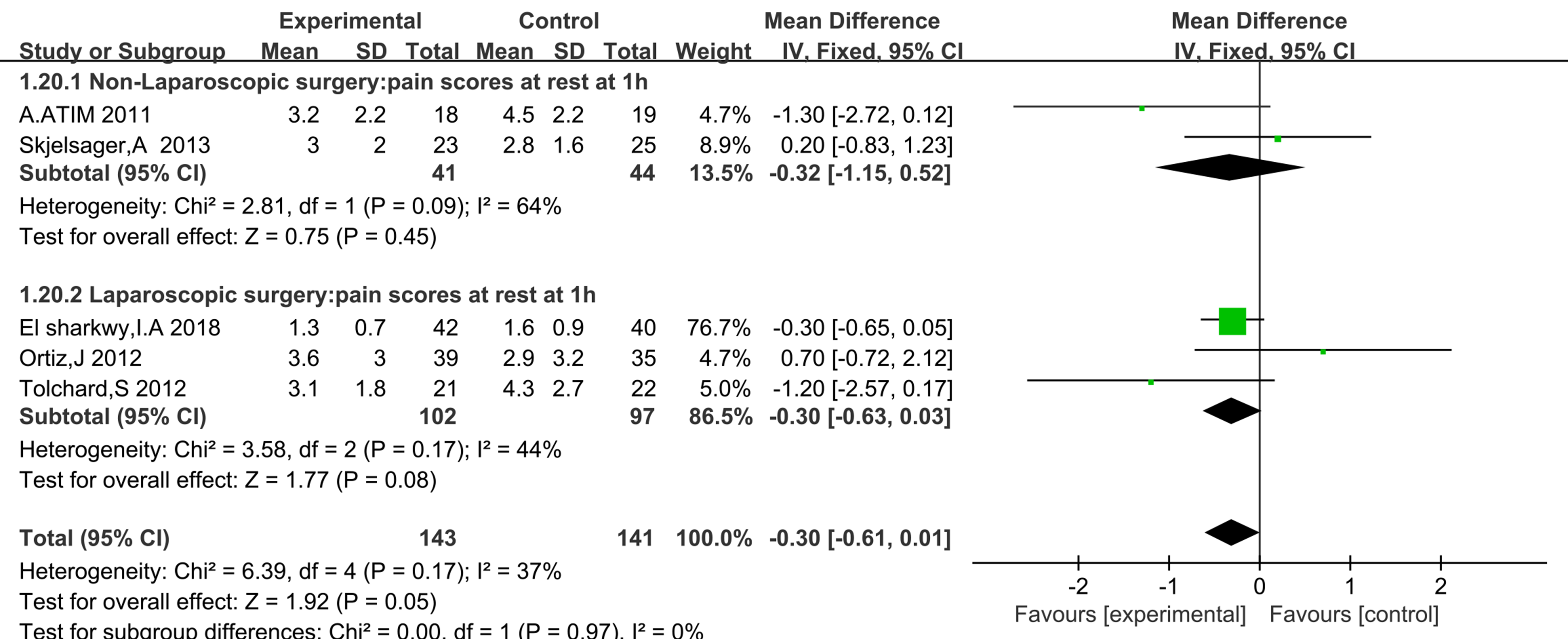

Supplement: Supplementary 2 — Fig S1: subgroup analysis of pain scores at rest at 1 h after nonlaparoscopic surgery VS laparoscopic surgery (TIF). [file 8914953.f2.tif]

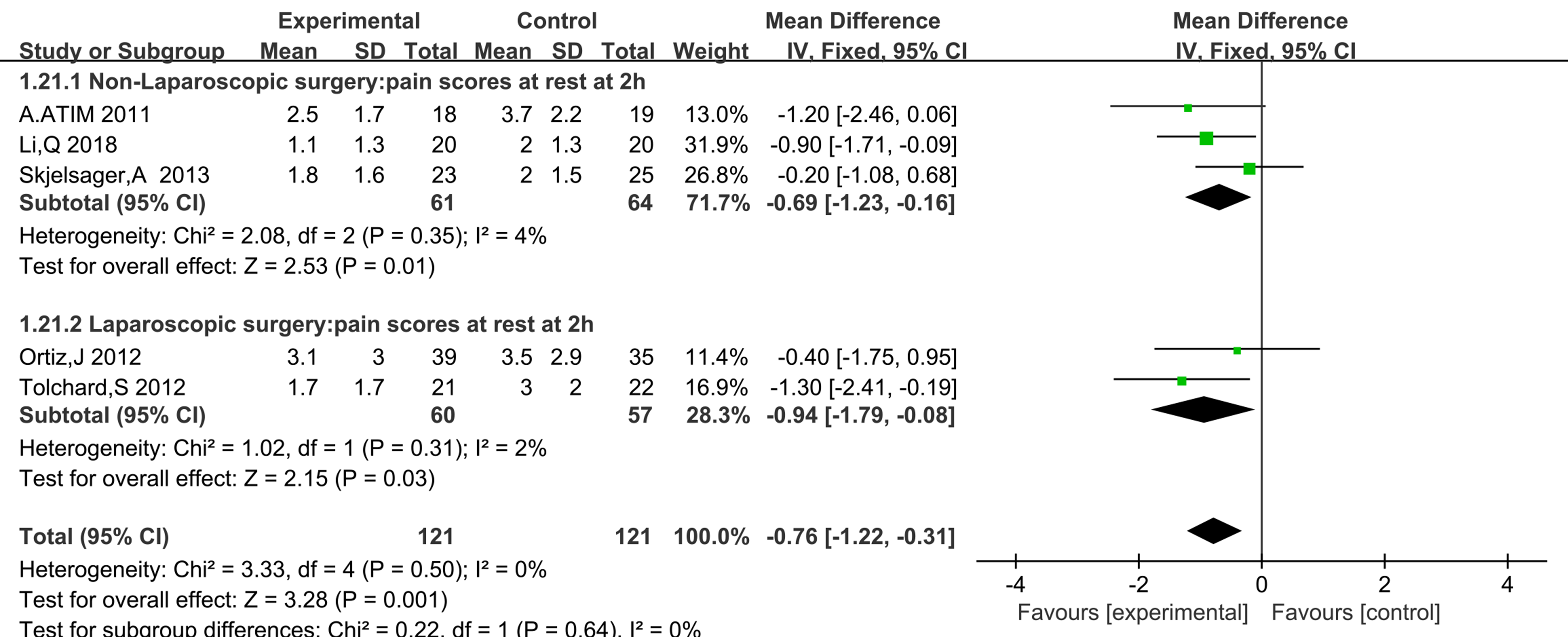

Supplement: Supplementary 3 — Fig S2: subgroup analysis of pain scores at rest at 2 h after nonlaparoscopic surgery VS laparoscopic surgery (TIF). [file 8914953.f3.tif]

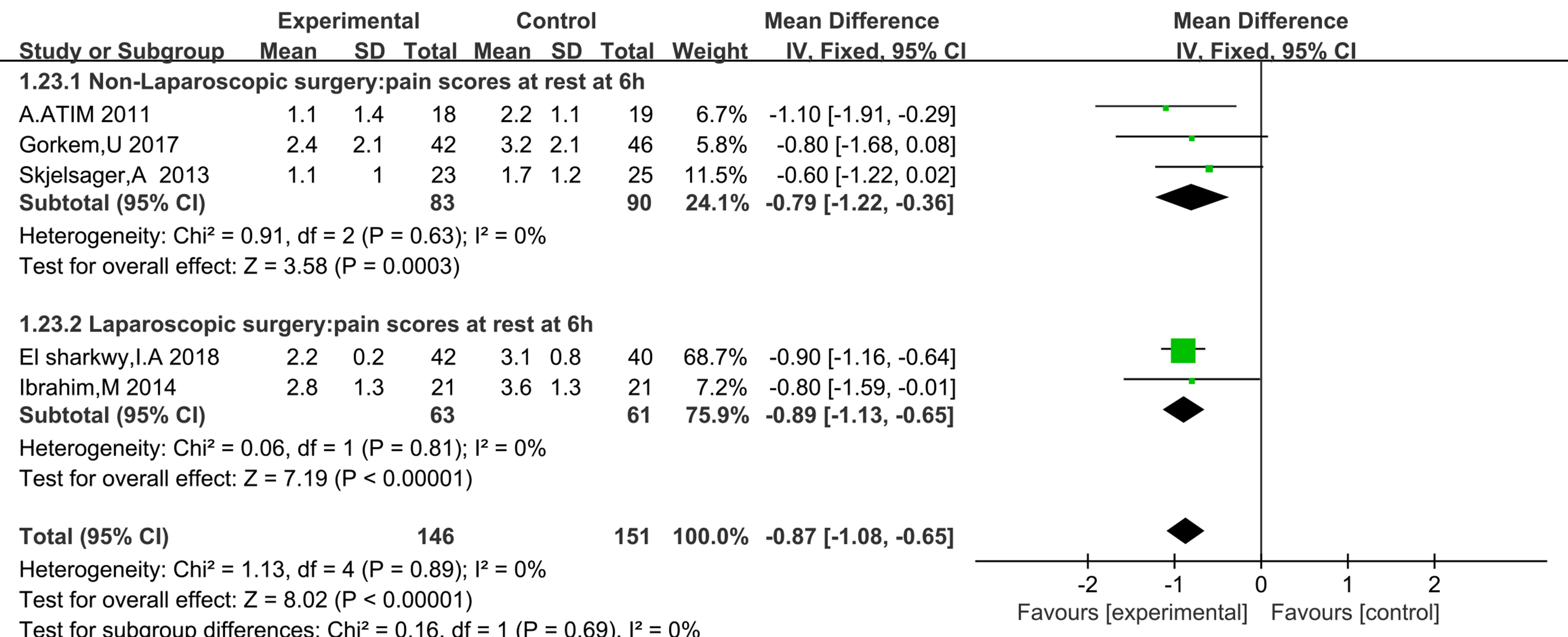

Supplement: Supplementary 4 — Fig S3: subgroup analysis of pain scores at rest at 6 h after nonlaparoscopic surgery VS laparoscopic surgery (TIF). [file 8914953.f4.tif]

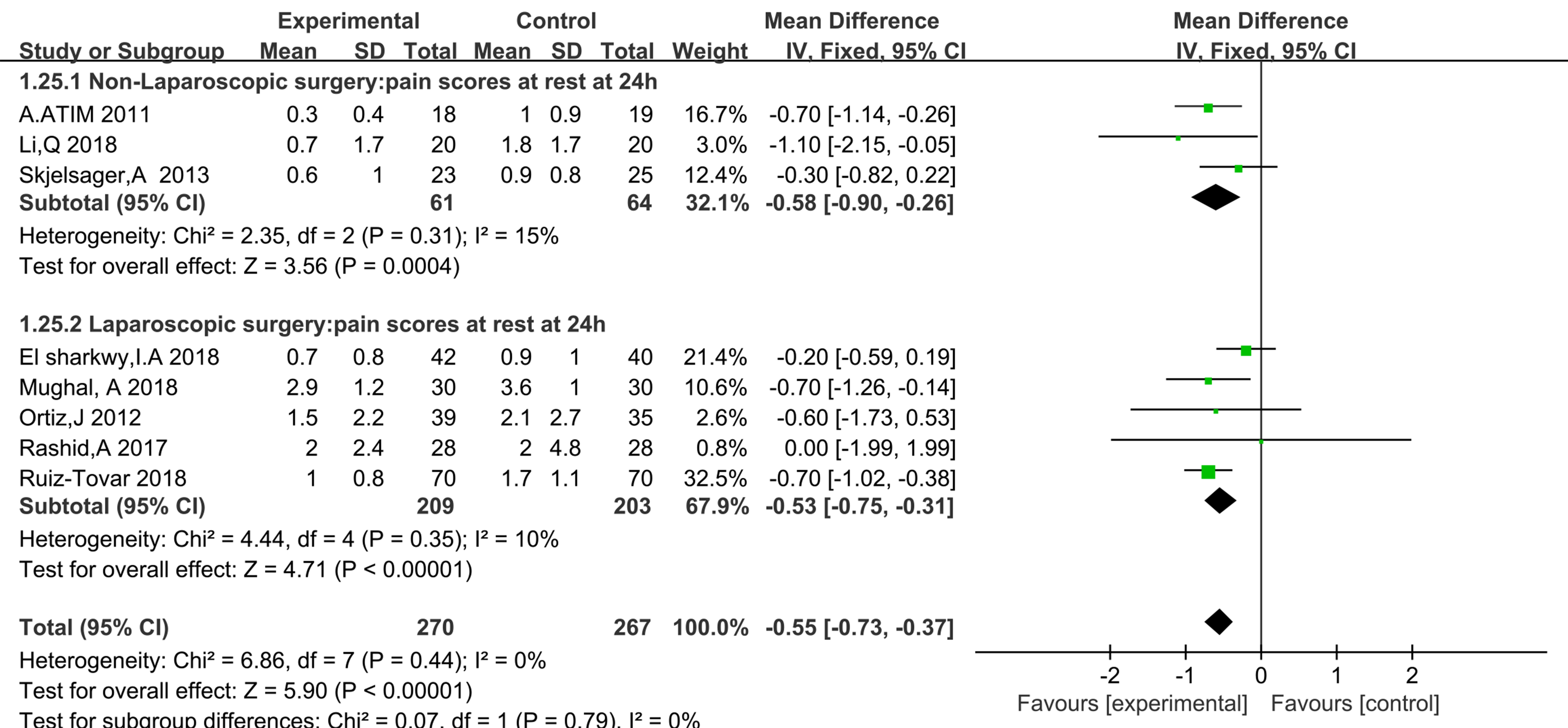

Supplement: Supplementary 5 — Fig S4: subgroup analysis of pain scores at rest at 24 h after nonlaparoscopic surgery VS laparoscopic surgery (TIF). [file 8914953.f5.tif]

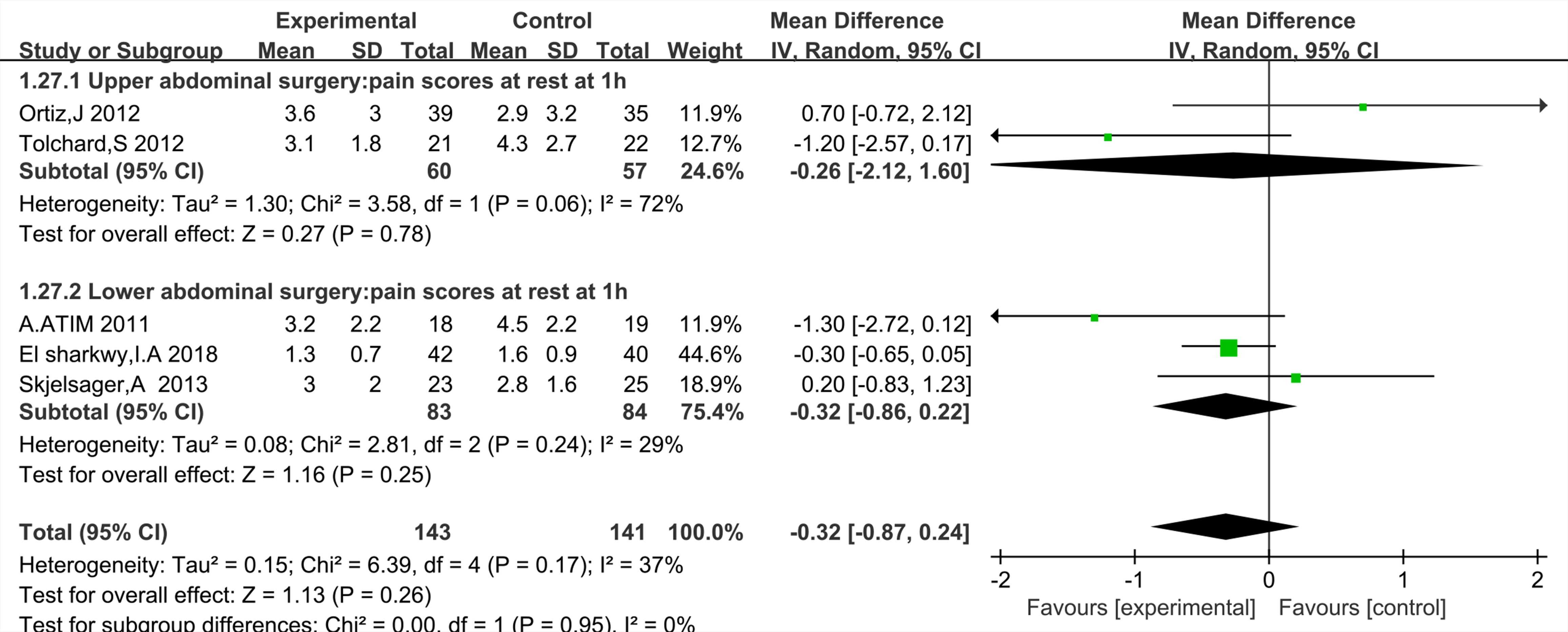

Supplement: Supplementary 6 — Fig S5: subgroup analysis of pain scores at rest at 1 h after upper abdominal surgery VS lower abdominal surgery (TIF). [file 8914953.f6.tif]

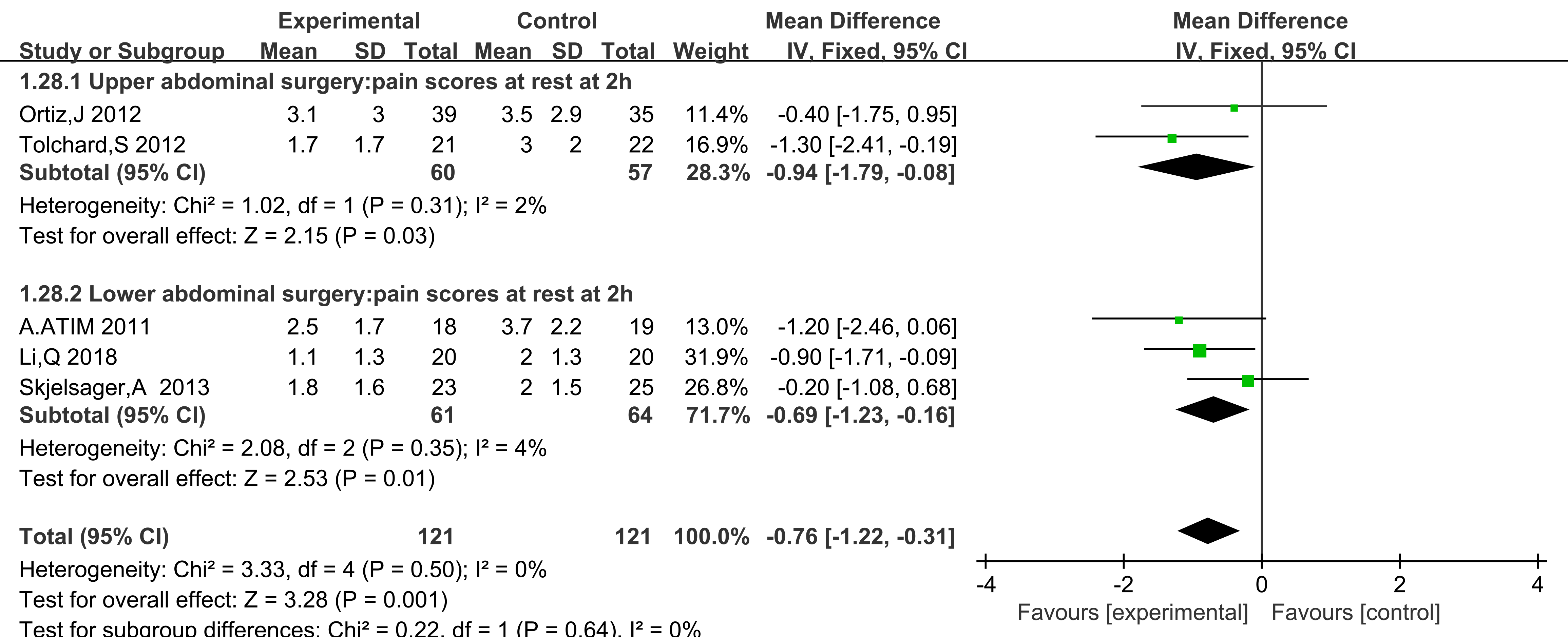

Supplement: Supplementary 7 — Fig S6: subgroup analysis of pain scores at rest at 2 h after upper abdominal surgery VS lower abdominal surgery (TIF). [file 8914953.f7.tif]

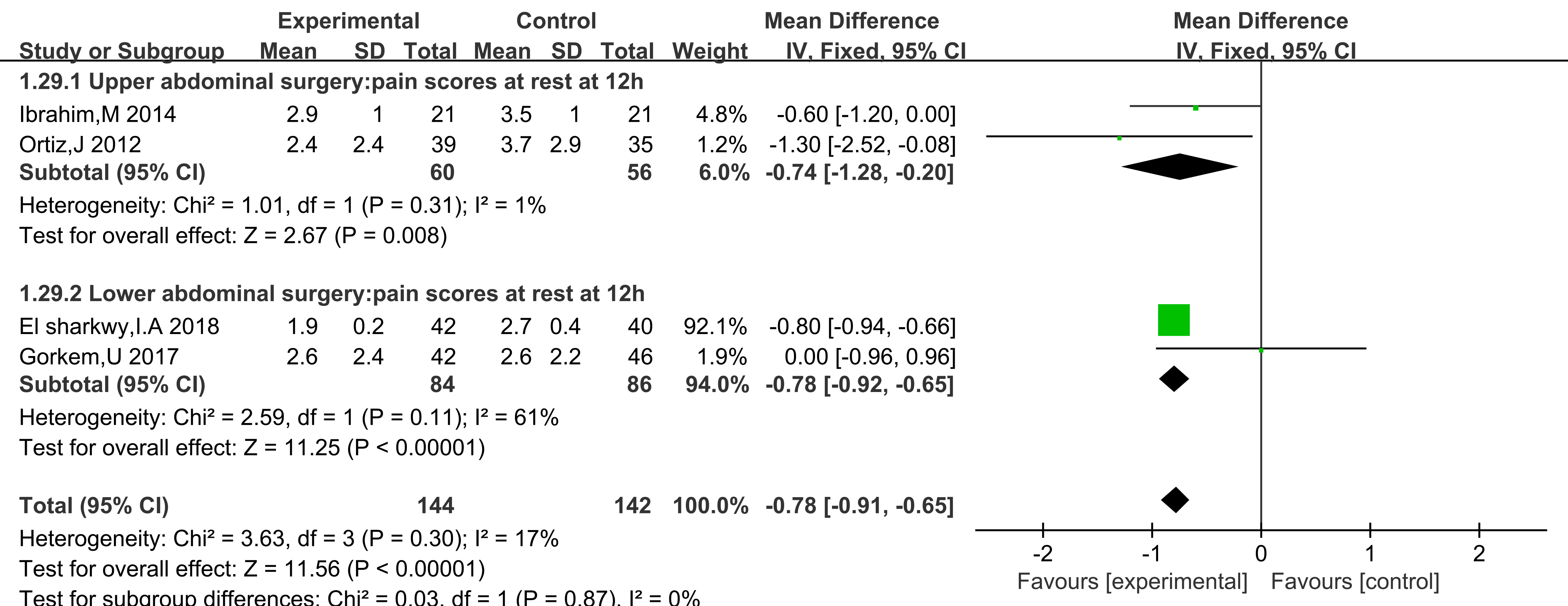

Supplement: Supplementary 8 — Fig S7: subgroup analysis of pain scores at rest at 12 h after upper abdominal surgery VS lower abdominal surgery (TIF). [file 8914953.f8.tif]

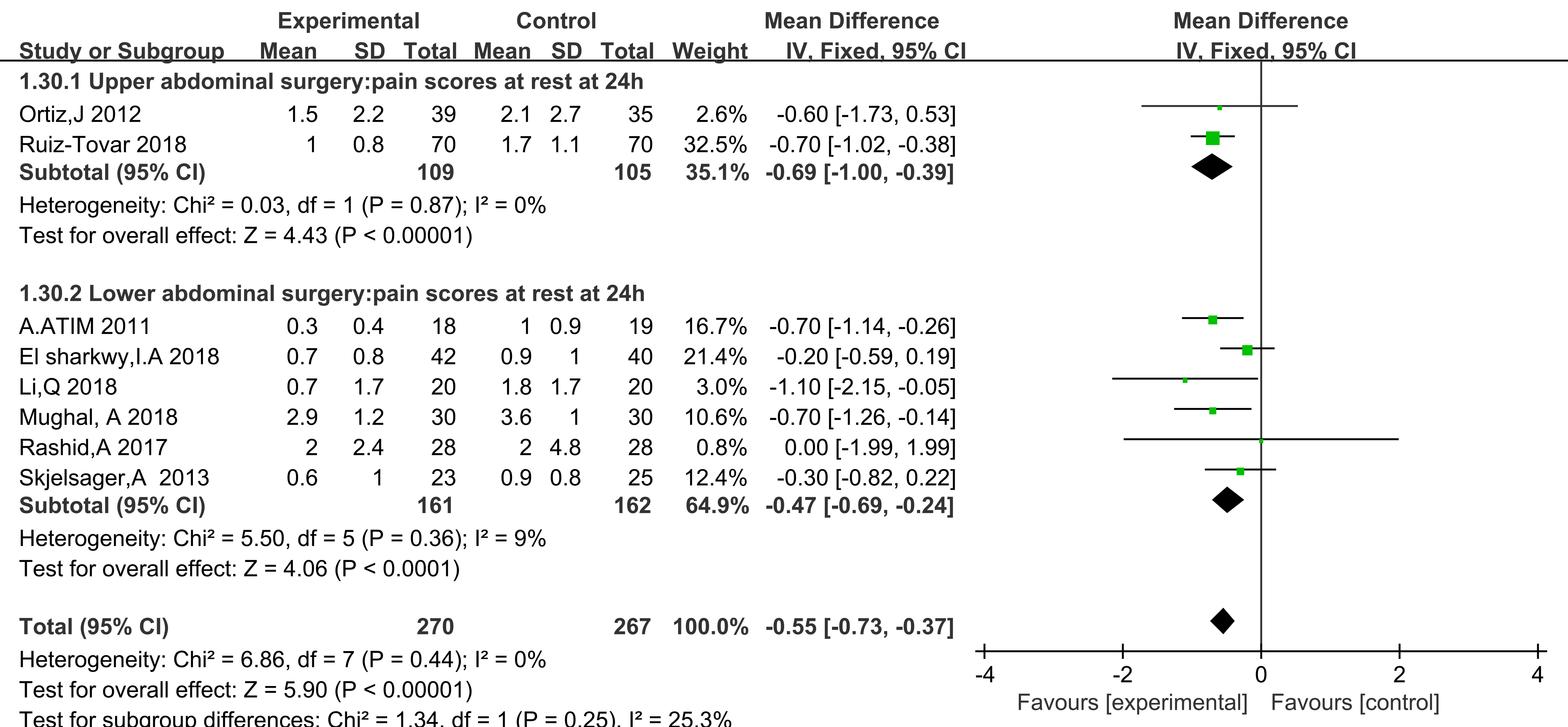

Supplement: Supplementary 9 — Fig S8: subgroup analysis of pain scores at rest at 24 h after upper abdominal surgery VS lower abdominal surgery (TIF). [file 8914953.f9.tif]
